# Unraveling the Central Proopiomelanocortin Neural Circuits

**DOI:** 10.3389/fnins.2013.00019

**Published:** 2013-02-22

**Authors:** Aaron J. Mercer, Shane T. Hentges, Charles K. Meshul, Malcolm J. Low

**Affiliations:** ^1^Department of Molecular and Integrative Physiology, University of MichiganAnn Arbor, MI, USA; ^2^Department of Biomedical Sciences, Colorado State UniversityFort Collins, CO, USA; ^3^Departments of Behavioral Neuroscience and Pathology, Oregon Health & Science UniversityPortland, OR, USA; ^4^Research Services, Neurocytology Laboratory, Veterans Affairs Medical CenterPortland, OR, USA; ^5^Department of Internal Medicine, Division of Metabolism, Endocrinology and Diabetes, University of MichiganAnn Arbor, MI, USA

**Keywords:** proopiomelanocortin neurons, hypothalamus, arcuate nucleus, energy homeostasis, neural networks, metabolism, obesity

## Abstract

Central proopiomelanocortin (POMC) neurons form a potent anorexigenic network, but our understanding of the integration of this hypothalamic circuit throughout the central nervous system (CNS) remains incomplete. POMC neurons extend projections along the rostrocaudal axis of the brain, and can signal with both POMC-derived peptides and fast amino acid neurotransmitters. Although recent experimental advances in circuit-level manipulation have been applied to POMC neurons, many pivotal questions still remain: how and where do POMC neurons integrate metabolic information? Under what conditions do POMC neurons release bioactive molecules throughout the CNS? Are GABA and glutamate or neuropeptides released from POMC neurons more crucial for modulating feeding and metabolism? Resolving the exact stoichiometry of signals evoked from POMC neurons under different metabolic conditions therefore remains an ongoing endeavor. In this review, we analyze the anatomical atlas of this network juxtaposed to the physiological signaling of POMC neurons both *in vitro* and *in vivo*. We also consider novel genetic tools to further characterize the function of the POMC circuit *in vivo*. Our goal is to synthesize a global view of the POMC network, and to highlight gaps that require further research to expand our knowledge on how these neurons modulate energy balance.

## Introduction

Proopiomelanocortin (POMC) neurons originating from the arcuate nucleus of the hypothalamus (ARC) are unequivocally important for maintaining metabolic homeostasis (Cone, 2005; Smart et al., 2006). The requirement of the central POMC neuronal network for energy balance has been established for almost 20 years, but precisely how an organism synthesizes and transmits information through hypothalamic POMC neurons remains enigmatic. This class of anorexigenic neurons is defined by the expression of the *Pomc* gene and its downstream peptide products: α-melanocyte stimulating hormone (α-MSH), β-MSH, γ-MSH, adrenocorticotropic hormone (ACTH), and β-endorphin (Mountjoy, 2010). α- and β-MSH are central to metabolic regulation through activation of the melanocortin 4 (MCR4) and MCR3 receptors, whereas γ-MSH is most potent at MCR3 (Roselli-Rehfuss et al., 1993). Agonism of these two receptors inhibits feeding behavior, promotes energy expenditure, and modulates energy partitioning (Roselli-Rehfuss et al., 1993; Harrold et al., 1999; Butler et al., 2000; Hwa et al., 2001). Furthermore, deficiencies in neural *Pomc* gene expression and melanocortin peptide production (Krude et al., 1998; Yaswen et al., 1999; Lee et al., 2006; Smart et al., 2006) or postsynaptic MCR function (Biebermann et al., 2006; Lee et al., 2008; Mencarelli et al., 2008) induce severe hyperphagia, reduced metabolic output, and weight gain.

The three primary central nervous system (CNS) opioids (β-endorphin, enkephalins, and dynorphins) are mixed agonists for μ-, κ-, and δ-opioid receptors. Stimulation of the central μ-opioid receptor system, in particular, by infusion of peptides including β-endorphin or synthetic agonists, is widely regarded to increase body mass and food intake, particularly of hedonically preferred macronutrients (Olszewski et al., 2011; DiFeliceantonio et al., 2012). Despite these actions of exogenously administered opioids, genetic ablation of β-endorphin from *Pomc* by insertion of a premature STOP codon results in a body weight increase of 10–15% due to increased fat storage in young male mice (Rubinstein et al., 1996; Low et al., 2003). Behavioral tests suggest that β-endorphin is a critical component of food reward circuitry (Hayward et al., 2002), but this opioid may also synergize with α-MSH to increase metabolic output and decrease food consumption long-term (Appleyard et al., 2003; Low et al., 2003). At the cellular level, β-endorphin can also directly modify POMC neuron activity by antagonizing the effects of α-MSH on downstream MCRs (Dutia et al., 2012) and by inhibiting POMC neurons through μ-opioid receptors (Pennock et al., 2012). Taken together, the peptide milieu released from POMC neurons is anorectic, but with a caveat: α-MSH is a direct suppressor of food intake, signaling through downstream MCRs, whereas β-endorphin, despite its apparent role in promoting hypophagia and fat metabolism, can directly temper POMC neuron and α-MSH activity. It is interesting to note, however, that the sensitivity to opioids is much greater at presynaptic μ-opioid receptors regulating POMC neurons than at the postsynaptic receptors (Pennock and Hentges, 2011). Therefore, since the majority of inputs to POMC neurons are inhibitory, it may be that endogenous opioids inhibit the presynaptic release of GABA onto POMC neurons, and thus disinhibit POMC neurons. Consistent with this possibility, recent work shows that optogenetically induced release of β-endorphin inhibits presynaptic transmitter release onto ARC AgRP neurons without causing a notable postsynaptic effect (Yang et al., 2011). Furthermore, a recent study demonstrated that dynorphin can directly inhibit the activity of POMC neurons via κ_2_ receptors located on the cell body and dendrites, while altering presynaptic tone via κ_1_ receptors located on axon terminals (Zhang and van den Pol, 2013).

Proopiomelanocortin neurons can be identified by virtue of *Pomc* gene and melanocortin or β-endorphin peptide expression. However, these cells exhibit diverse anatomical connections and neurochemical profiles. The dendrites, presumptive sites of synaptic inputs in addition to the POMC cell somas, currently remain largely intractable to analysis. Emerging evidence also indicates that subpopulations of POMC neurons have the capacity to signal with both neuropeptides and amino acid neurotransmitters (Hentges et al., 2004, 2009; Dicken et al., 2012) and that these hypothalamic neuroendocrine cells integrate signals at sites distal to the ARC (Hentges, 2007). Taken together, POMC neurons have a tremendous capacity for integrating peripheral and central metabolic cues, while releasing both peptides and amino acid transmitters throughout the CNS. In this review, we examine the heterogeneous population of POMC neurons to synthesize a more complete picture of this circuit. To close, we consider recent advances in transgenic mouse lines as well as circuit-level analysis with new tools such as optogenetics and designer receptors exclusively activated by designer drugs (DREADD) technology. These advanced technologies are essential to further elucidate the function of this network in the context of energy balance, as significant gaps still exist regarding POMC anatomy and physiology in the CNS. A more complete understanding of the POMC neural network is critical to determine its function in obesity and related metabolic disorders.

## Light Microscopic Anatomy of the Central POMC System

The primary population of CNS POMC-expressing neurons consists of a few thousand cells spanning the entire rostrocaudal extent of the ARC and within the periarcuate region. In *Mus musculus*, there are ∼3,000 total POMC neurons in the mediobasal hypothalamus (Cowley et al., 2001; Plum et al., 2009) that extend fibers to multiple nuclei throughout the CNS (Cone, 2005; Hentges, 2007; King and Hentges, 2011; Lim et al., 2012). This distribution parallels the anatomy of POMC cell bodies and fibers identified on the basis of peptidergic immunoreactivity in the rat, human, and macaque brain (Desy and Pelletier, 1978; Jacobowitz and O’Donohue, 1978; Watson et al., 1978; Khachaturian et al., 1984). For consistency, our discussion of the ultrastructural morphology of POMC neurons will focus on observations in the rat, whereas modern approaches in genetics, anatomy, and physiology described in this review will primarily focus on our understanding of the POMC circuit in mice.

Predominant CNS regions containing POMC peptide immunoreactive fibers include nuclei implicated in feeding (ARC, paraventricular nucleus of the hypothalamus (PVH), lateral hypothalamus, and nucleus tractus solitarius (NTS); Cone, 2005; Zheng et al., 2010), reward (bed nucleus of the stria terminalis, nucleus accumbens, septal nucleus, ventral tegmental area, and central amygdala; Arvaniti et al., 2001; Pandit et al., 2011; Lim et al., 2012), and analgesia (periaqueductal gray, dorsal raphe nucleus; Chen et al., 1992; Wang et al., 1998). These axonal projections parallel the patterns of MCR (Gantz et al., 1993; Roselli-Rehfuss et al., 1993; Mountjoy et al., 1994) and opioid receptor (Minami and Satoh, 1995) expression, suggesting a relatively tight pre- and postsynaptic juxtaposition of POMC peptide release sites to MCR- or μ-opioid expressing effector cells. An additional small population of ∼300 POMC neurons is found in the NTS (Palkovits and Eskay, 1987; Joseph and Michael, 1988). Although it has been suggested that NTS POMC neurons assist in integrating gut and CNS feeding signals (Joseph et al., 1983; Corp et al., 1998; Grill et al., 1998), selective ablation of the ventral hypothalamus reduces POMC-positive fiber immunoreactivity in the brainstem by over two-thirds (Kitahama et al., 1986; Joseph and Michael, 1988; Barna et al., 1997; Zheng et al., 2010) and thus, POMC projections emanating from the ARC appear to be the principal signaling unit for *Pomc*-derived peptides throughout the CNS in adult animals.

## Ultrastructural Organization of POMC Neurons

Ultrastructural analyses of POMC neurons described cell bodies 20–30 μm in diameter, with prominent organelles for peptide synthesis (Bugnon et al., 1981; Lamberts and Goldsmith, 1985) including an abundant rough endoplasmic reticulum and extensive Golgi complexes that extended from the perinuclear zone to the axon hillock. It should be noted that the anatomy characterized in these studies was performed on animals pre-treated with colchicine, resulting in an increased cytoplasmic accumulation of small, clear vesicles, and dense core granules. While this technique allows for neurochemical identification at the level of the perikarya, it does not resolve the location of endogenous release sites. However, in these studies, golgi complexes exhibited a close association with both dense core granules and vesicles, indicating that, at the ultrastructural level, POMC neurons might have the capacity to release both amino acid neurotransmitters and secretory peptides. Pre-embedding immunoelectron microscopy revealed that both small clear vesicles and dense core granules traffic to the axons and synapses of POMC neurons identified in the ARC (Matsumoto and Arai, 1978; Kiss and Williams, 1983; Buma et al., 1989). Other neurosecretory systems also have the capacity to release peptides from the soma or dendritic arbors (Morris and Pow, 1991; Huang and Neher, 1996; Ludwig et al., 2002), but these mechanisms appear to be cell-type specific (Ramamoorthy et al., 2011). POMC neurons indeed have large, clear vesicles present in dendritic branches (Leranth et al., 1980), but whether these vesicles are utilized for the dendritic release of peptides or amino acid neurotransmitters has not been determined.

Unidentified neurons originating in the ARC contain an average of two or three dendrites, which are spatially oriented dependent on the proximity of the soma to the third ventricle (van den Pol and Cassidy, 1982). Dendrites from medial neurons are oriented in the dorsoventral axis, while lateral neurons preferentially project mediolaterally. Moreover, ARC neurons characteristically have thin, tapering dendrites instead of a dense, branched dendritic plexus. Axons from ARC neurons either terminate at synapses on other local neurons, or bifurcate and send one collateral within the ARC and extend a second fiber to another nucleus (van den Pol and Cassidy, 1982). POMC neurons recapitulate this generalized description: multiple thin dendrites are found on POMC neurons within the ARC, while tapered, elongated processes extend hundreds of μm along the dorsoventral axis (Hentges, 2007). Although dendrites are considered the traditional “input” branch of a cell, POMC neurons are also synaptically innervated directly on their cell somas (Horvath et al., 1992, 2010; Guan et al., 2008). Axon terminals from POMC neurons exist both within the ARC near POMC somas (Matsumoto and Arai, 1978; Chen and Pelletier, 1983; Cowley et al., 2001; Hentges et al., 2004, 2009), and also at distal sites throughout the diencephalon and mesencephalon (Zheng et al., 2010; King and Hentges, 2011). Thus, both inputs to and outputs from POMC neurons occur locally within the ARC as well as some distance from the ARC.

## Central Inputs to POMC Neurons

Techniques utilized in the handful of studies examining POMC dendrites have been limited by several drawbacks. The Golgi staining technique (van den Pol and Cassidy, 1982) provided robust labeling, but the authors were unable to specifically identify POMC neurons, and they did not examine distal processes outside of the ARC. Similarly, analyses of POMC neurons labeled intracellularly with neurobiotin (Hentges, 2007) are limited by dialysis of the biotin solution and the fact that cells in tissue slices will have some of their distal processes cut during slice preparation. Nonetheless, both approaches indicate the relative lack of spines on dendritic processes, which makes the identification of POMC dendrites more difficult (Liu et al., 2012). Therefore, our knowledge of the dendritic organization of POMC neurons is far from complete.

Comparatively, the established anatomical dogma for hypothalamic gonadotropin-releasing hormone (GnRH) neurons is that of a relatively simple bipolar morphology with one or two dendrites. Recently, Herde et al. (2011) showed that a subpopulation of GnRH neurons have highly branched dendrites that extend beyond the blood-brain barrier (BBB) into the organum vasculosum of the lamina terminalis (OVLT). In this region, GnRH neurons can directly sense molecules circulating in the bloodstream, and the branched dendritic network can putatively provide a large surface area for efficient sampling. ARC POMC neurons, on the other hand, are located just dorsal to the median eminence, sending fibers throughout its entirety (Kiss et al., 1985). Similar to the OVLT, the fenestrated microcapillaries surrounding the median eminence (Golding, 1994; Meister, 2007; Norsted et al., 2008) may allow POMC fibers, including both axons and dendrites, to directly transmit and receive signals outside of the CNS. These new findings in GnRH neurons advocate parallel studies of POMC dendrites to better understand the integration of metabolic signals through POMC networks.

Peptidergic and amino acid neurotransmitter inputs driving POMC neuronal activity have been studied from the level of animal behavior down to single cell electrophysiological responses, and it is therefore out of the scope of this review to cover the literature on this subject in its entirety. Likewise, the effects of peripheral hormones such as leptin and insulin on central POMC neurons have been recently reviewed at length (Morton and Schwartz, 2011; Williams et al., 2011). Instead, we will focus on the primary CNS circuits that impinge upon POMC neurons.

### AgRP/NPY/GABA neurons

The classic antagonistic circuit to POMC neurons includes a separate population of cells in the ARC, the neuropeptide-Y/agouti-related peptide (NPY/AgRP) neurons. NPY/AgRP neurons release three transmitters reported to stimulate feeding: AgRP, NPY, and γ-aminobutyric acid (GABA; Wu and Palmiter, 2011). AgRP was initially discovered to be a potent inhibitor of MCR function (Ollmann et al., 1997), and was subsequently characterized as an inverse agonist of MC3R and MC4R activity *in vitro* and *in vivo* (Shutter et al., 1997; Haskell-Luevano and Monck, 2001; Nijenhuis et al., 2001; Tolle and Low, 2008). However, a second *in vivo* experiment suggested that AgRP might function only as a competitive MCR antagonist without inverse agonist actions (Corander et al., 2011). In these studies, double-knockout mice lacking both *Pomc* and *Agrp (Pomc^−/−^;Agrp^−/−^*) recapitulated the obesogenic phenotype observed in *Pomc^−/−^* single knockouts, questioning the ability of CNS AgRP to promote an orexigenic phenotype independently of endogenous melanocortin peptides. Further pharmacological approaches in *Pomc^−/−^*; *Agrp^−/−^* mice indicated that exogenous AgRP could antagonize the effects of α-MSH on feeding, but AgRP peptide alone did not enhance the already increased feeding behavior of these mice. Along the same lines, AgRP may also negatively shift the constitutive activity of the MCRs, or induce the endocytosis of target MCRs (Breit et al., 2006). A final consensus on the mechanism of action by AgRP on MCRs is still being debated (Flier, 2006), but AgRP nonetheless appears to negatively regulate downstream MCRs. In addition to inhibitory effects on MCRs, AgRP, and GABA release from AgRP/NPY neurons can evoke a hyperpolarizing response directly on ARC POMC neurons (Smith et al., 2007; Tong et al., 2008). NPY also hyperpolarizes POMC neurons through the neuropeptide-Y receptor 1-activation of G-protein inwardly rectifying potassium channels (Roseberry et al., 2004). At the histochemical and ultrastructural level, AgRP neurons impinge upon the perikarya of POMC neurons throughout the ARC (Horvath et al., 1992, 2010; Newton et al., 2013), and these neurons can directly inhibit POMC neurons through the synaptic release of GABA (Atasoy et al., 2012). Thus, AgRP neurons can negatively modulate POMC function by a direct GABAergic synaptic mechanism and indirectly by antagonizing postsynaptic MCRs.

Although NPY exhibits diverse functions throughout the brain (Redrobe et al., 2002; Dube, 2007), AgRP neurons exhibit >99% co-localization with NPY in the ARC. While the orexigenic function of AgRP and NPY peptides is well documented (Ollmann et al., 1997; Hollopeter et al., 1998; Rossi et al., 1998), further studies have probed which transmitter released by AgRP/NPY neurons is critical for the positive modulation of feeding behavior. Constitutive knockouts of *Agrp* and *Npy* genes or downstream NPY receptors in mice failed to produce feeding or body weight deficits, and these mice responded normally with compensatory increased feeding behavior in response to food-restriction (Palmiter et al., 1998; Qian et al., 2002; Thorsell and Heilig, 2002; Corander et al., 2011). Knock-in mice harboring *Npy* alleles with a tetracycline-off (Tet-Off) regulatory domain and a minimal promoter inserted into exon 1 were developed to examine the necessity of NPY to promote feeding behavior (Ste Marie et al., 2005). The mutant allele produced a fourfold constitutive elevation in *Npy* mRNA, while administration of doxycycline greatly reduced the levels of *Npy* mRNA and NPY protein measured in the whole brain of these mice compared to control animals. Conspicuously, these functional gene dosage manipulations also failed to induce phenotypic alterations in body weight homeostasis (Ste Marie et al., 2005). It has been suggested that in the cases of early developmental ablation of NPY/AgRP neurons, gradual adult depletion of NPY/AgRP neurons, or gradual reduction in levels of NPY peptides, the CNS circuitry makes compensatory adaptations for the loss of signaling from these neurons (Ste Marie et al., 2005; Pierce and Xu, 2010; Wu and Palmiter, 2011).

A persuasive body of evidence has accumulated that GABA released from NPY/AgRP neurons is the critical transmitter modulating feeding behavior, as the severe anorectic phenotype induced by the acute ablation of AgRP neurons by diptheria toxin in adult mice can be rescued with chronic infusion of a benzodiazepine partial agonist (Wu et al., 2008, 2009). Along the same lines, selective deletion of the GABA/Glycine-specific vesicular transporter VGAT from AgRP neurons results in lean mice that are resistant to obesity (Tong et al., 2008). Despite the antagonistic effects of AgRP on MCRs, it is hypothesized that the inhibitory effects of GABA may be the primary mechanism by which AgRP neurons counter the POMC network throughout the CNS. While GABA release from AgRP neurons can directly inhibit POMC neurons, it also inhibits neurons in the parabrachial nucleus (PBN), which is a relay center that integrates information about feeding, nausea, and taste aversion (Wu et al., 2012a).

An alternative explanation for the orexigenic nature of NPY/AgRP neurons, based on recent optogenetic experiments, is that these neurons primarily target oxytocin neurons within the PVH to enhance feeding behavior (Atasoy et al., 2012). Oxytocin neurons are direct targets of both NPY/AgRP and POMC neurons due to their expression of MCRs (Sabatier et al., 2003), and therefore NPY/AgRP neurons can inhibit oxytocin neurons by a combination of MCR antagonism and GABA transmission. However, depleting central oxytocin neurons postnatally elicits minimal effects on feeding behavior, and instead results in animals that are only prone to high fat diet-induced obesity (Wu et al., 2012b). Despite these disparate findings, AgRP neurons may impinge both on oxytocin neurons to inhibit energy expenditure (Wu et al., 2012b) and promote feeding (Atasoy et al., 2012), while reinforcing the hedonic component of feeding by signaling in the PBN (Wu et al., 2012a). While questions remain regarding the detailed mechanisms of AgRP neuron actions, technological advances in circuit-level manipulations of transgenic mice reinforce that AgRP neuron activation results in positive energy balance, positing that AgRP neurons are a primary circuit in the CNS that opposes many functions of ARC POMC neurons (Aponte et al., 2011; Krashes et al., 2011).

### Serotonergic neurons

In the late twentieth century, the drug fenfluramine was utilized as an appetite suppressant. Fenfluramine stimulates the central serotonergic (5-HT) system, which led to potent weight loss effects in patients (Halford et al., 2011). Problematically, the drug had off-target effects in the cardiovascular system, and its use as a weight loss medication was discontinued in the mid-to-late 1990s. Nonetheless, the effects of fenfluramine on the CNS were further probed in animal models, and it was revealed that the effects of 5-HT stimulation resulted in the activation of POMC neurons in the ARC (Heisler et al., 2002; Giorgetti and Tecott, 2004). Global knockout of the 5-HT_2C_ receptor (5-HT_2C_R) induced pronounced hyperphagia, hyperactivity, and obesity in mice, but selective re-expression of 5-HT_2C_Rs in POMC neurons resulted in the normalization of body composition, feeding behavior, and peripheral insulin sensitivity (Xu et al., 2008, 2010a). These results were further extended to downstream MCR-expressing neurons, and it was found that fenfluramine stimulated POMC neurons to release melanocortin peptides onto cognate MC3R and MC4R throughout the CNS (Lam et al., 2008; Rowland et al., 2010; Xu et al., 2010b).

The exact intracellular mechanism by which 5-HT_2C_Rs induce a depolarization of POMC neurons is unclear. 5-HT_2C_Rs are G_q_ G-protein coupled receptors (GPCRs) that generally stimulate neurons through phospholipase C intracellular cascades (Lam et al., 2010). In this cascade, activated phospholipase C cleaves phosphatidylinositol 4,5-bisphosphate into diacyl glycerol and inositol 1,4,5-bisphosphate (IP_3_). Among other functions, IP_3_ can stimulate the release of intracellular Ca^2+^ stores in the endoplasmic reticulum, which can result in increased granule and vesicle release. It has also been reported that serotonin activates transient receptor potential channels (TRPC; Sohn et al., 2011), similar to leptin signaling (Qiu et al., 2010). Others have shown that serotonin blunts the function of potassium channels, which act to hyperpolarize the cell’s resting membrane potential, in POMC neurons (Qiu et al., 2007; Roepke et al., 2012). Taken together, after 5-HT_2C_R activation these mechanisms can all induce a depolarizing response and lead to the firing of action potentials by POMC neurons.

The entire serotonergic circuit originates from the dorsal and ventral raphe nuclei, but the primary inputs to ARC POMC neurons appear to project from the B7 subnucleus of the dorsal raphe (Lam and Heisler, 2007). Similar to the phenotypic heterogeneity observed in insulin- versus leptin-sensitive POMC neurons (Williams et al., 2010), individual POMC neurons have been reported to be activated by leptin or serotonin, but not both ligands (Sohn et al., 2011). In these studies, the authors mapped the activity of POMC neurons in the ARC based on a given transmitter stimulus. Serotonin-sensitive neurons lay more medial and rostral in the ARC, whereas leptin-sensitive neurons map to more laterocaudal portions of the ARC. Insulin-sensitive POMC neurons are distributed similarly to serotonin-sensitive POMC neurons, but it is unclear if these two populations are anatomically distinct or if they can respond to both effectors. This heterogeneity, however, begets larger questions: are subpopulations of POMC neurons more critical than others to modulate feeding behavior and energy expenditure? Do these different populations exert different metabolic effects? Selective deletion of leptin and insulin receptors from POMC neurons resulted in only a mild obesogenic phenotype (Balthasar et al., 2004; Hill et al., 2010), whereas a global knockout and subsequent POMC-specific rescue of 5-HT_2C_R expression normalized mice that were initially hyperphagic and obese (Xu et al., 2008). Despite tools to specifically manipulate receptor expression in POMC neurons, leptin, serotonin, and insulin are all regarded to exert potent effects on feeding behavior, energy expenditure, and glucose homeostasis (Hill et al., 2010; Williams et al., 2011). Thus, corroborating the diverse POMC population with other CNS circuits and peripheral targets is an ongoing effort in the field of metabolism research.

## POMC Synapses

To release bioactive signaling molecules, POMC neurons integrate afferent signals until a depolarization induces the release of peptidergic vesicles and granules in a calcium-dependent manner (O’Donohue et al., 1981; Bunel et al., 1990). In *ex vivo* brain slices, POMC neurons exhibit a canonical pathway to release peptidergic granules: the opening of voltage-gated sodium channels propagates an action potential which results in an influx of Ca^2+^ through voltage-gated calcium channels to release α-MSH-containing granules (Tiligada and Wilson, 1988; Tranchand Bunel et al., 1989). Basal release of POMC peptides, however, appears to operate independently of depolarization-induced Ca^2+^ influx (Jegou et al., 1987), possibly by capitalizing on intracellular stores of Ca^2+^ within the endoplasmic reticulum. At the synapse, Ca^2+^ presumably induces SNARE machinery to intertwine with surface glycoproteins on synaptic vesicles and granules to release peptides and transmitters into the synaptic cleft. Although POMC neurons are typically characterized by the expression and release of POMC-derived neuropeptides, almost all POMC neurons express the cocaine- and amphetamine-regulated transcript (*Cart*) gene and encoded CART peptide (Elias et al., 1998) and ∼30% also show co-localization with prodynorphin immunoreactivity (Maolood and Meister, 2008; Zhang and van den Pol, 2013).

### POMC-derived peptides and peptide-receptor mismatch

As α-MSH, β-endorphin, and ACTH are all POMC*-*derived peptide products, the axonal fibers, and synaptic terminals of central POMC neurons exhibit unequivocal expression of these three peptides across species (O’Donohue and Dorsa, 1982; Chen and Pelletier, 1983; Kiss and Williams, 1983; Dores and Baron, 2011). Because fibers immunoreactive for POMC peptides co-ramify in target nuclei expressing either MCRs or opioid receptors, a simplistic scheme would suggest that POMC boutons and presynaptic terminals directly release neuropeptides onto other neurons expressing the appropriate receptors. However, neuroendocrine circuits such as the POMC system exhibit an interesting paradox: peptides released from POMC neurons can be detected at sites devoid of postsynaptic receptors (MacMillan et al., 1998), and at the ultrastructural level, neurons within the hypothalamus including presumptive POMC neurons appear to have the machinery to release peptides at non-synaptic sites as well (Leranth et al., 1980; Buma and Nieuwenhuys, 1988; Buma et al., 1989; van Lookeren Campagne et al., 1991). It has been suggested that circuits like the central POMC system may participate in both synaptic and volume transmission in the CNS (Golding, 1994; Agnati et al., 1995; van den Pol, 2012). Although POMC neurons form classically described synaptic contacts with postsynaptic dendritic fibers (Bugnon et al., 1981), they also exocytose neuropeptides into the ventricular circulation. POMC fibers straddle the cerebral ventricles along the rostrocaudal extent of the brain (Cone, 2005), and it has been demonstrated that POMC and POMC-derived peptides circulate throughout the ventricular network, possibly to act on distal target sites irrespective of a direct POMC synaptic contact (Kaye et al., 1987; Tsigos et al., 1993; Veening et al., 2012). This idea is recapitulated by the fact that intracerebroventricular administration of melanocortin agonists reduces feeding and body weight in experimental animals (Murphy et al., 1998, 2000), whereas melanocortin antagonists applied by the same route induce hyperphagia and weight gain (Rossi et al., 1998; Irani et al., 2011). In contrast to this view, focal injections of melanocortin agonists in the hypothalamus (Giraudo et al., 1998; Kim et al., 2000) or brainstem (Grill et al., 1998; Williams et al., 2000; Sutton et al., 2005; Zheng et al., 2005) reduce food intake and promote weight loss, suggesting that POMC neurons indeed have specified effector targets that, at least in part, are required to maintain metabolic homeostasis.

Proopiomelanocortin peptides may also be released at non-synaptic sites to act as paracrine signals. For example, chronic neuropathic pain is associated with phosphorylation and desensitization of μ-opioid receptors in the dorsal striatum of wild-type mice. These receptor events are blocked in β-endorphin knockout mice, but retained in proenkephalin or prodynorphin knockout mice, suggesting that β-endorphin is the endogenous opioid peptide that physiologically interacts with striatal μ-receptors in this experimental paradigm (Petraschka et al., 2007). However, there are no POMC or β-endorphin immunoreactive fibers in the striatum of wild-type mice indicating that the source of β-endorphin must be at some distance from its target requiring diffusion of the peptide from its site of release, possibly via the cerebrospinal fluid.

### GABA and glutamate release from POMC neurons

Ultrastructural observations of POMC synapses revealed large dense core granules that were immunopositive for POMC peptides, but also small, clear vesicles localized to synaptic terminals (Kiss et al., 1984; Buma et al., 1989). The morphological properties of POMC vesicles matched that of vesicles in synapses throughout the CNS. Electron microscopic measurements of such vesicles are described as small, clear compartments with a diameter of ∼50 nm concentrated at active zones in presynaptic terminals (Heuser, 1989). The central POMC circuit has been described to have subpopulations of cells that are glutamatergic or cholinergic (Ovesjo et al., 2001; Collin et al., 2003; Meister et al., 2006; Meister, 2007), but these studies were limited to immunohistochemical descriptions of the ARC.

We developed an *in vitro* culture system of neurons expressing fluorophores under control of *Pomc* transgenes that allowed an electrophysiological characterization of identified neurons (Hentges et al., 2004, 2009). Cultured primary neurons maintained for 2 weeks prior to experimentation formed autaptic synapses, which proved useful for single neuron unit dissection of the amino acid neurotransmitter phenotypes in these cells. Patch-clamp recordings revealed both GABAergic and glutamatergic subpopulations of POMC neurons. In acute brain slices, electrophysiological, and channelrhodopsin-based stimulation of POMC neurons induced evoked release of both GABA and glutamate (Dicken et al., 2012; Pennock et al., 2012) indicating the ability of these neurons to release amino acid transmitters in an intact circuit and excluding the possibility of artifactual results from the earlier *in vitro* experiments. Consistent with GABA release, ∼40% of POMC neurons express the GABA-synthesizing enzymes GAD67 and GAD65 (Hentges et al., 2004; Jarvie and Hentges, 2012). To load synaptic vesicles with GABA, the canonical VGAT molecule traverses the vesicle membrane and loads GABA in a proton-dependent manner (Gasnier, 2004). Furthermore, a smaller fraction of POMC neurons express the vesicular glutamate transporter VGLUT2 (Collin et al., 2003; Vong et al., 2011; Jarvie and Hentges, 2012), consistent with the finding of glutamate release from some POMC neurons.

A dissenting view regarding the existence of GABAergic POMC neurons was published recently: neurons in the CNS expressing a tdTomato marker following Cre-recombinase mediated deletion of a loxP-flanked stop signal by a VGAT-Cre transgene did not overlap with *Pomc-hrGFP* neurons (Vong et al., 2011). However, POMC neurons express the enzymes to synthesize GABA (Hentges et al., 2004; Jarvie and Hentges, 2012) and the synaptic machinery to release GABAergic vesicles in isolated POMC cultures (Hentges et al., 2004, 2009) and in acute brain slices (Dicken et al., 2012). Because POMC neurons do not appear to express standard VGAT isoforms, it is likely that they use an unidentified transporter protein or an alternative mechanism to load vesicles with GABA. Dopaminergic neurons in the substantia nigra pars compacta can release GABA in a VGAT-independent manner by instead loading GABA into vesicles using the vesicular monoamine transport protein 2 (VMAT2; Tritsch et al., 2012). These researchers also discovered that VMAT2 is sufficient to restore GABA loading and release in *Vgat^−/−^* neurons. A similar mechanism may load GABA into vesicles in POMC neurons, but to date this has not been examined.

At the ultrastructural level in *M. musculus*, POMC presynaptic terminals contain both POMC peptides and amino acid neurotransmitters (C. K. Meshul and M. J. Low, unpublished observations). Using transgenic mice expressing enhanced GFP (EGFP) under control of the *Pomc* promoter (Cowley et al., 2001), axon terminals from POMC neurons were identified by pre-embedding immunohistochemistry and electron microscopy following the methods of Meshul and McGinty (Meshul et al., 1994, 1999; Meshul and McGinty, 2000). POMC neurons were identified using primary antisera to GFP or ACTH and visualized using biotinylated secondary antibodies and an avidin-coupled DAB reaction product, whereas GABA and glutamate were detected at EGFP- and ACTH-positive synaptic terminals with immunogold-coupled antibodies.

Proopiomelanocortin axon terminals were readily identified as GABAergic or glutamatergic in these preparations. Bona fide POMC terminals were identified based on their combination of electron-dense DAB reaction product, content of synaptic vesicles and granules, and the presence of characteristic synaptic plasma membrane specializations at the juxtaposition of the terminal to a postsynaptic dendritic process or soma. GABA or glutamate immunogold-labeled presynaptic terminals formed symmetric or asymmetric synaptic contacts to postsynaptic neurons, respectively. These terminals contained >10 gold particles per μm^2^, determined to be the minimum concentration of labeled transmitter particles to identify positively labeled axon terminals above background signal. Qualitatively, GABA-positive nerve terminals exhibited the greater density of immunoreactive amino acid neurotransmitter in POMC-positive nerve terminals and were identified more readily in ultra-thin sections, consistent with the finding that ∼40% of the central POMC circuit is GABAergic (Jarvie and Hentges, 2012). Glutamatergic POMC neuron terminals on the other hand, were much rarer, but nevertheless expressed glutamate in association with synaptic vesicles.

Interestingly, GABAergic and glutamatergic POMC neurons combined appear to comprise only 50% of the entire network. *Gad65* and *Gad67* mRNAs are expressed in predominately overlapping populations of POMC neurons, therefore about 50% of the POMC neurons in the ARC lack detectable markers indicative of GABA or glutamate phenotypes under basal conditions (S. T. Hentges, unpublished observation). The possibility that more POMC neurons could be identified as GABAergic or glutamatergic under different conditions, such as when energy balance is perturbed, has not yet been explored. In addition to GABAergic and glutamatergic phenotypes, immunohistochemical evidence suggests that a few POMC cells may be cholinergic (Meister et al., 2006). We have not observed cholinergic POMC neurons using *in situ* hybridization or electrophysiological approaches, and it remains to be determined if cholinergic markers co-localize with amino acid transmitters in POMC neurons. It is possible that a subpopulation of POMC neurons may have no additional neurochemical phenotype, releasing only POMC and CART peptides. However, this possibility will be difficult to definitively demonstrate since identifying the diverse neurochemical phenotypes at POMC synapses may be hampered by amino acid transmitter levels that are below the threshold of detection for many standard neuroanatomical techniques.

## Physiologic Consequences of Activating POMC Neurons

In a recent study, Aponte et al. (2011) activated POMC or AgRP networks using channelrhodopsin-based photostimulation. Photostimulation of AgRP neurons induced a robust hyperphagic response within 1 h, whereas activation of POMC neurons using the same technology required a 24-h stimulation to dampen feeding in transgenic mice. Although *in vivo* evidence suggests a much more prolonged time scale for a POMC-dependent decrease in appetitive behavior, *ex vivo* brain slice recordings show that channelrhodopsin expressing POMC neurons can release an opioid (presumably β-endorphin) in response to acute photostimulation (Yang et al., 2011). The evidence that β-endorphin can act in an auto-inhibitory fashion on presynaptic POMC neurons may at least partially explain the delay in feeding cessation in optically stimulated POMC neurons (Pennock et al., 2012). The work by Yang et al. postulates that POMC neurons can inhibit presynaptic glutamatergic inputs to AgRP/NPY neurons in an opioidergic fashion, but it is unclear how robustly this mechanism affects AgRP/NPY neurons *in vivo*. The slice preparation experiments indicate that POMC neurons, however, can directly affect the tone of a counter-regulatory ARC circuit by an evoked stimulus. Despite the rapid actions noted in the slice preparation, the *in vivo* optogenetic studies suggest varied possibilities for POMC peptide signaling: (1) a significant stimulus over time is required to elicit a response on feeding behavior, (2) POMC peptides diffuse slowly to their targets in the CNS, (3) that activation of POMC neurons may affect food intake preferentially under certain conditions, such as at the onset of the dark cycle when POMC neurons are usually inhibited, or (4) that very specific patterns of stimulation are required to adequately recapitulate the firing patterns of POMC neurons *in vivo*. To date it is unclear what physiological stimuli regulate the spatiotemporal release of peptides versus amino acid transmitters, and therefore it remains to be seen how different combinations of neurotransmitters and peptides affect postsynaptic neurons. For example, POMC neurons may release α-MSH to activate a postsynaptic MCR-expressing cell, but this effect may be dampened if a POMC presynaptic terminal co-releases GABA. Along the same lines, a central infusion of β-endorphin can blunt the anorectic effects of centrally applied α-MSH (Dutia et al., 2012) despite the fact that both endogenous peptides originate from POMC neurons and presumably are co-released from POMC projections. While all POMC neurons express α-MSH, β-endorphin, and ACTH, we do not know if these peptides are present in equal proportions in all POMC fibers throughout the brain. Thus, POMC neurons may modulate postsynaptic neurons differentially depending on their explicit neurochemical profile.

## Stoichiometry and Plasticity of the Central POMC Circuit: Perspectives and Future Directions

Proopiomelanocortin dendrites, considered the classical sites of peptidergic and amino acid integration, remain largely unstudied in POMC neurons. Despite the availability of antisera developed against classic dendritic markers such as postsynaptic density 95 and the microtubule associated proteins, these markers have not been identified in POMC neurons. Therefore, it is difficult to deduce both the neurochemical phenotypes and anatomical origins of neurons impinging upon POMC cells. In theory, Cre-dependent expression of dendritic markers specifically in POMC-Cre neurons may reveal the sites of input to POMC neurons, but this will depend on the capacity of these cells to express and target transgenic proteins specifically to dendritic projections. In addition to classical dendritic inputs, evidence from the Horvath laboratory suggests that POMC neurons are modulated by presynaptic neurons directly at the level of the perikarya (Pinto et al., 2004), which is consistent with chronic nutritional or hormonal changes altering the axo-dendritic and axo-somatic contacts in the arcuate nucleus (Parducz et al., 2003; Csakvari et al., 2007; Horvath et al., 2010). These works present a few intriguing possibilities regarding the synaptic inputs to the POMC circuit, primarily that somatic inputs, rather than dendritic inputs, may contribute significantly to the function of POMC neurons. Likewise, treating *ob/ob* mice with leptin was able to attenuate increases in inhibitory synaptic inputs to POMC neurons. The electrophysiological data combined with electron microscopic data implicating rapid shifts in the wiring from presynaptic nerve terminals onto POMC cell bodies over the course of a few hours suggest an unconventional mode of regulation of POMC neuron activity. However, it is unclear if these somatic inputs are a primary driver of POMC neuron function. The mechanism underlying these rapid alterations in connectivity has not been fully described.

Furthermore, although the electron microscopic evidence is compelling, the corresponding electrophysiological data presented by Pinto et al. (2004) do not completely parallel the anatomic rearrangements and do not rule out the likelihood that altered synaptic activity may be more heavily driven at dendritic sites that were not studied using electron microscopy techniques. The presence of synaptic inputs to POMC neurons at distal dendrites has been demonstrated in studies showing that retrograde regulation of POMC neurons by endocannabinoids occurs at GABAergic synapses impinging on POMC dendrites considerably rostral to the ARC (Hentges, 2007), emphasizing that these cells indeed process dendritic inputs at sites distal to the ARC. A possible explanation for the “rewiring” of the circuit may be a shift in POMC dendrite size or dynamic changes in GABA or glutamate receptor expression on the surface of POMC neurons, similar to the fasting-induced plasticity of glutamate receptors observed in the AgRP/NPY circuit (Liu et al., 2012). Nevertheless, there is growing impetus to parse out how the wiring in the hypothalamus, including POMC neurons, can dynamically change when an organism’s diet or disease state is dramatically shifted (Zeltser et al., 2012). Because the central POMC circuit is regarded as a primary anorexigenic driver, one would expect decreases in POMC neural activity in a fasted state, enhanced firing in a fed state, and according to findings from the Horvath group (Pinto et al., 2004; Horvath et al., 2010), decreased inhibitory inputs to POMC neurons in *ob/ob* mice that are treated with leptin. The exact time scale of this synaptic plasticity and the dysregulation of this circuit in obesity and other maladies are not known.

On the other hand, if we consider the functional segregation of the POMC circuit based on three prototypically studied inputs, the network can also be divided into leptin-, insulin-, or serotonin-sensitive POMC neurons. Parsing out the respective sensitivity of different subpopulations of POMC neurons to each of these inputs suggests that individual POMC neurons are only sensitive to leptin, insulin, or serotonin (Williams et al., 2010; Sohn et al., 2011), but to date a handful of POMC neurons are reported to respond to both insulin and leptin (Al-Qassab et al., 2009). The entire population of POMC neurons in the mouse CNS totals only ∼3000 cells, and respective hormone and peptide receptors likely exist in a dynamic state of fluctuation in terms of expression, turnover, and desensitization, which makes determining the total number of neurons sensitive to a given signal a significant challenge. To date it is unclear if all POMC neurons express receptors for one of these inputs or how the contributions of amino acid transmitter receptor activation versus peptide signaling bear on downstream POMC function. Thus, the breadth of how metabolic cues induce the plasticity of the POMC network has not been fully deduced, and it remains to be determined how these putative morphological changes alter different subpopulations of POMC neurons.

At the axon terminals, many POMC neurons release the amino acid neurotransmitters GABA and glutamate, whereas all POMC axonal fibers appear to ubiquitously express the neuropeptides ACTH, α-MSH, and β-endorphin by immunohistochemical techniques. However, it is not known if peptide and non-peptide transmitters are released equally from all fibers. Additionally, many POMC axons have boutons *en passant* that may release peptides from dense core granules at non-synaptic targets (Buma and Nieuwenhuys, 1988; Buma et al., 1989; van Lookeren Campagne et al., 1991). These findings indicate that we still do not have a full grasp on how opiomelanocortin-derived peptides are released from POMC neurons, or if these peptides are released equivalently from all cells. Furthermore, there may be distinct anatomical correlations between POMC subtypes and their CNS effector regions, but this has not been explicitly determined. If we consider the phenotypic differences of both the signal integration and peptide release from the central POMC circuit, there are at least a dozen possible subpopulations of POMC neurons in the ARC. An acute or chronic change in nutritional status would be likely to significantly alter the synaptic output of subpopulations of the POMC circuit. For example, the satiety hormone leptin may stimulate a subset of POMC neurons to predominantly release glutamate and α-MSH, subsequently reducing food intake and increasing an animal’s metabolic output. Thus, POMC target sites throughout the CNS may experience a cocktail of contrasting inputs from the POMC circuit depending on which phenotypes of cells are activated in relation to the feeding status of an animal.

Taken together, the integrative aspects of POMC peptide and synaptic communication are still unclear despite this circuit’s well-established role in energy balance. Although it is enticing to reduce the output of the POMC circuit to a small region of the brain, it is more likely that the POMC network affects multiple targets throughout the CNS with a dynamic synergy. Future studies using advanced tools to image fluorophore-labeled POMC peptides at the molecular level may reveal the function of this circuit in living brain tissue. At this level, peptide synthesis, trafficking, and release could be monitored in real-time in both acute brain slices and in live animals. Current reductionist approaches to dissect the POMC circuit seek to parse out the dendritic receptors and synaptic transmitters expressed by POMC neurons required to maintain body composition. In many instances these studies employ Cre/Lox technology to alter the phenotype of the POMC circuit. Problematically, currently available *Pomc-Cre* transgenic mouse lines activate floxed transgenes in both mature POMC neurons and cells that express *Pomc* only transiently during development (Padilla et al., 2010, 2012). A second *Pomc-Cre* line was developed in the lab of Dr. Gregory Barsh (Xu et al., 2005a,b). The initial characterization of these mice suggested that only 2% of Cre-positive neurons in the ARC were not POMC neurons. However, we conducted an independent cell lineage tracing analysis of this alternative *Pomc-Cre* line crossed to the ROSA26-LacZ reporter strain and counted ∼8,000 β-galactosidase positive neurons per ARC, a number well in excess of the normally detectable 3,000 immunopositive POMC neurons in an adult mouse (M. J. Low and A. J. Mercer, unpublished observations). Furthermore, there was extensive reporter expression in many other non-ARC hypothalamic nuclei, the zona incerta, and additional extrahypothalamic nuclei similar to the pattern previously reported (Padilla et al., 2010, 2012) using the *Pomc-Cre* line created by Dr. Brad Lowell.

To overcome these developmental issues, researchers have been employing virally mediated, Cre-dependent expression of channelrhodopsin proteins in the POMC neurons of adult animals (Aponte et al., 2011; Yang et al., 2011; Dicken et al., 2012). Cre expression in adult *Pomc-Cre* mice appears to be restricted solely to mature POMC neurons, and thus allows for a more accurate analysis of the POMC circuit. Activation of channelrhodopsin proteins by light pulses delivered through a guided cannula *in vivo* or optically on *ex vivo* brain slices produces depolarizing membrane potentials, thus allowing for exogenous control of the POMC circuit (Tye and Deisseroth, 2012). As discussed previously, optogenetic stimulation of POMC neurons *in vivo* appears to require a significant amount of time and activation to induce a change in feeding behavior (Aponte et al., 2011), whereas evoked release of β-endorphin or the amino acid neurotransmitters GABA and glutamate can be accomplished within milliseconds or seconds in an acute brain slice preparation (Yang et al., 2011; Dicken et al., 2012).

Alternatively, DREADD technology is likely to be capitalized on for future studies of the POMC circuit (Dong et al., 2010). Virally mediated, Cre-dependent expression of DREADD receptors can be controlled by synthetic ligands to activate either G_q_ or G_i_ signaling cascades specifically in POMC-Cre neurons. Similar to channelrhodopsin-mediated stimulation of AgRP neurons, activating G_q_-coupled DREADDs in AgRP neurons can induce a potent upregulation of feeding behavior (Krashes et al., 2011). While channelrhodopsin activation leads to a change in the membrane voltage of a stimulated neuron, DREADD expressing cells will activate GPCR cascades instead. Together, both technologies offer artificial mechanisms for directly controlling a single population of CNS neurons, and eliminate the possibility of off-target pharmacological effects using intracerebroventricular drug administration or bath application of drugs on an acute brain slice. In addition, these technologies may allow us to uncover the molecular mechanisms governing amino acid neurotransmitter and POMC peptide release to determine their respective contributions to POMC-driven anorexia. Deducing the nature of how the POMC network signals throughout the brain is of critical importance, as this circuit represents a potential pharmacotherapeutic target to ameliorate the persistent burden of obesity and its related metabolic complications.

## Conflict of Interest Statement

The authors declare that the research was conducted in the absence of any commercial or financial relationships that could be construed as a potential conflict of interest.

## References

[B1] Agnati L. F. Zoli M. Stromberg I. Fuxe K. (1995). Intercellular communication in the brain: wiring versus volume transmission. Neuroscience 69, 711–726 10.1016/0306-4522(95)00308-6 8596642

[B2] Al-Qassab H. Smith M. A. Irvine E. E. Guillermet-Guibert J. Claret M. Choudhury A. I. (2009). Dominant role of the p110beta isoform of PI3K over p110alpha in energy homeostasis regulation by POMC and AgRP neurons. Cell Metab. 10, 343–354 10.1016/j.cmet.2009.09.008 19883613 PMC2806524

[B3] Aponte Y. Atasoy D. Sternson S. M. (2011). AGRP neurons are sufficient to orchestrate feeding behavior rapidly and without training. Nat. Neurosci. 14, 351–355 10.1038/nn.2739 21209617 PMC3049940

[B4] Appleyard S. M. Hayward M. Young J. I. Butler A. A. Cone R. D. Rubinstein M. (2003). A role for the endogenous opioid beta-endorphin in energy homeostasis. Endocrinology 144, 1753–1760 10.1210/en.2002-221096 12697680

[B5] Arvaniti K. Huang Q. Richard D. (2001). Effects of leptin and corticosterone on the expression of corticotropin-releasing hormone, agouti-related protein, and proopiomelanocortin in the brain of ob/ob mouse. Neuroendocrinology 73, 227–236 10.1159/000054639 11340336

[B6] Atasoy D. Betley J. N. Su H. H. Sternson S. M. (2012). Deconstruction of a neural circuit for hunger. Nature 488, 172–177 10.1038/nature11270 22801496 PMC3416931

[B7] Balthasar N. Coppari R. Mcminn J. Liu S. M. Lee C. E. Tang V. (2004). Leptin receptor signaling in POMC neurons is required for normal body weight homeostasis. Neuron 42, 983–991 10.1016/j.neuron.2004.06.004 15207242

[B8] Barna I. Koenig J. I. Makara G. B. (1997). Effects of anterolateral and posterolateral cuts around the medial hypothalamus on the immunoreactive ACTH and beta-endorphin levels in selected brain regions of the rat. Brain Res. Bull. 42, 353–357 10.1016/S0361-9230(96)00298-5 9092876

[B9] Biebermann H. Castaneda T. R. Van Landeghem F. Von Deimling A. Escher F. Brabant G. (2006). A role for beta-melanocyte-stimulating hormone in human body-weight regulation. Cell Metab. 3, 141–146 10.1016/j.cmet.2006.01.007 16459315

[B10] Breit A. Wolff K. Kalwa H. Jarry H. Buch T. Gudermann T. (2006). The natural inverse agonist agouti-related protein induces arrestin-mediated endocytosis of melanocortin-3 and -4 receptors. J. Biol. Chem. 281, 37447–37456 10.1074/jbc.M605982200 17041250

[B11] Bugnon C. Bloch B. Lenys D. (1981). Ultrastructural study of presumptive pro-opiocortin producing neurons in the rat hypothalamus. Neuroscience 6, 1299–1313 10.1016/0306-4522(81)90189-5 6167898

[B12] Buma P. Nieuwenhuys R. (1988). Ultrastructural characterization of exocytotic release sites in different layers of the median eminence of the rat. Cell Tissue Res. 252, 107–114 10.1007/BF00213831 3378255

[B13] Buma P. Veening J. Nieuwenhuys R. (1989). Ultrastructural characterization of adrenocorticotrope hormone (ACTH) immunoreactive fibres in the mesencephalic central grey substance of the rat. Eur. J. Neurosci. 1, 659–672 10.1111/j.1460-9568.1989.tb00372.x 12106124

[B14] Bunel D. T. Delbende C. Blasquez C. Jegou S. Vaudry H. (1990). Effects of ions and ionic channel activators or blockers on release of alpha-MSH from perifused rat hypothalamic slices. Brain Res. Mol. Brain Res. 8, 167–175 10.1016/0169-328X(90)90061-H 1698247

[B15] Butler A. A. Kesterson R. A. Khong K. Cullen M. J. Pelleymounter M. A. Dekoning J. (2000). A unique metabolic syndrome causes obesity in the melanocortin-3 receptor-deficient mouse. Endocrinology 141, 3518–3521 10.1210/en.141.9.3518 10965927

[B16] Chen J. Rao Z. R. Shi J. W. (1992). The sites of origin of a-melanocyte-stimulating hormone-containing axonal components in the lateral area of the midbrain periaqueductal gray of the rat. Brain Res. 575, 333–336 10.1016/0006-8993(92)90100-N 1571791

[B17] Chen Y. Y. Pelletier G. (1983). Demonstration of contacts between proopiomelanocortin neurons in the rat hypothalamus. Neurosci. Lett. 43, 271–276 10.1016/0304-3940(83)90200-8 6324045

[B18] Collin M. Backberg M. Ovesjo M. L. Fisone G. Edwards R. H. Fujiyama F. (2003). Plasma membrane and vesicular glutamate transporter mRNAs/proteins in hypothalamic neurons that regulate body weight. Eur. J. Neurosci. 18, 1265–1278 10.1046/j.1460-9568.2003.02840.x 12956725

[B19] Cone R. D. (2005). Anatomy and regulation of the central melanocortin system. Nat. Neurosci. 8, 571–578 10.1038/nn1455 15856065

[B20] Corander M. P. Rimmington D. Challis B. G. O’Rahilly S. Coll A. P. (2011). Loss of agouti-related peptide does not significantly impact the phenotype of murine POMC deficiency. Endocrinology 152, 1819–1828 10.1210/en.2010-1450 21363936 PMC3137464

[B21] Corp E. S. Conze D. B. Smith F. Campfield L. A. (1998). Regional localization of specific [125I]leptin binding sites in rat forebrain. Brain Res. 789, 40–47 10.1016/S0006-8993(97)01547-3 9602048

[B22] Cowley M. A. Smart J. L. Rubinstein M. Cerdan M. G. Diano S. Horvath T. L. (2001). Leptin activates anorexigenic POMC neurons through a neural network in the arcuate nucleus. Nature 411, 480–484 10.1038/35078085 11373681

[B23] Csakvari E. Hoyk Z. Gyenes A. Garcia-Ovejero D. Garcia-Segura L. M. Parducz A. (2007). Fluctuation of synapse density in the arcuate nucleus during the estrous cycle. Neuroscience 144, 1288–1292 10.1016/j.neuroscience.2006.11.008 17161546

[B24] Desy L. Pelletier G. (1978). Immunohistochemical localization of alpha-melanocyte stimulating hormone (alpha-MSH) in the human hypothalamus. Brain Res. 154, 377–381 10.1016/0006-8993(78)90709-6 99212

[B25] Dicken M. S. Tooker R. E. Hentges S. T. (2012). Regulation of GABA and glutamate release from proopiomelanocortin neuron terminals in intact hypothalamic networks. J. Neurosci. 32, 4042–4048 10.1523/JNEUROSCI.6032-11.2012 22442070 PMC3321840

[B26] DiFeliceantonio A. G. Mabrouk O. S. Kennedy R. T. Berridge K. C. (2012). Enkephalin surges in dorsal neostriatum as a signal to eat. Curr. Biol. 22, 1918–1924 10.1016/j.cub.2012.08.014 23000149 PMC3482294

[B27] Dong S. Rogan S. C. Roth B. L. (2010). Directed molecular evolution of DREADDs: a generic approach to creating next-generation RASSLs. Nat. Protoc. 5, 561–573 10.1038/nprot.2009.239 20203671

[B28] Dores R. M. Baron A. J. (2011). Evolution of POMC: origin, phylogeny, posttranslational processing, and the melanocortins. Ann. N. Y. Acad. Sci. 1220, 34–48 10.1111/j.1749-6632.2010.05928.x 21388402

[B29] Dube C. (2007). Neuropeptide Y: potential role in recurrent developmental seizures. Peptides 28, 441–446 10.1016/j.peptides.2006.10.017 17196709 PMC1852447

[B30] Dutia R. Meece K. Dighe S. Kim A. J. Wardlaw S. L. (2012). beta-Endorphin antagonizes the effects of alpha-MSH on food intake and body weight. Endocrinology 153, 4246–4255 10.1210/en.2012-1166 22778225 PMC3423622

[B31] Elias C. F. Lee C. Kelly J. Aschkenasi C. Ahima R. S. Couceyro P. R. (1998). Leptin activates hypothalamic CART neurons projecting to the spinal cord. Neuron 21, 1375–1385 10.1016/S0896-6273(00)80656-X 9883730

[B32] Flier J. S. (2006). AgRP in energy balance: will the real AgRP please stand up? Cell Metab. 3, 83–85 10.1016/j.cmet.2006.01.003 16459309

[B33] Gantz I. Konda Y. Tashiro T. Shimoto Y. Miwa H. Munzert G. (1993). Molecular cloning of a novel melanocortin receptor. J. Biol. Chem. 268, 8246–8250 8463333

[B34] Gasnier B. (2004). The SLC32 transporter, a key protein for the synaptic release of inhibitory amino acids. Pflugers Arch. 447, 756–759 10.1007/s00424-003-1091-2 12750892

[B35] Giorgetti M. Tecott L. H. (2004). Contributions of 5-HT(2C) receptors to multiple actions of central serotonin systems. Eur. J. Pharmacol. 488, 1–9 10.1016/j.ejphar.2004.01.036 15044029

[B36] Giraudo S. Q. Billington C. J. Levine A. S. (1998). Feeding effects of hypothalamic injection of melanocortin 4 receptor ligands. Brain Res. 809, 302–306 10.1016/S0006-8993(98)00837-3 9853124

[B37] Golding D. W. (1994). A pattern confirmed and refined – synaptic, nonsynaptic and parasynaptic exocytosis. Bioessays 16, 503–508 10.1002/bies.950160710 7945279

[B38] Grill H. J. Ginsberg A. B. Seeley R. J. Kaplan J. M. (1998). Brainstem application of melanocortin receptor ligands produces long-lasting effects on feeding and body weight. J. Neurosci. 18, 10128–10135 9822766 10.1523/JNEUROSCI.18-23-10128.1998 PMC6793290

[B39] Guan J. L. Okuda H. Takenoya F. Kintaka Y. Yagi M. Wang L. (2008). Synaptic relationships between proopiomelanocortin- and ghrelin-containing neurons in the rat arcuate nucleus. Regul. Pept. 145, 128–132 10.1016/j.regpep.2007.09.028 17936371

[B40] Halford J. C. Boyland E. J. Lawton C. L. Blundell J. E. Harrold J. A. (2011). Serotonergic anti-obesity agents: past experience and future prospects. Drugs 71, 2247–2255 10.2165/11596680-000000000-00000 22085383

[B41] Harrold J. A. Widdowson P. S. Williams G. (1999). Altered energy balance causes selective changes in melanocortin-4(MC4-R), but not melanocortin-3 (MC3-R), receptors in specific hypothalamic regions: further evidence that activation of MC4-R is a physiological inhibitor of feeding. Diabetes 48, 267–271 10.2337/diabetes.48.2.267 10334300

[B42] Haskell-Luevano C. Monck E. K. (2001). Agouti-related protein functions as an inverse agonist at a constitutively active brain melanocortin-4 receptor. Regul. Pept. 99, 1–7 10.1016/S0167-0115(01)00234-8 11257308

[B43] Hayward M. D. Pintar J. E. Low M. J. (2002). Selective reward deficit in mice lacking beta-endorphin and enkephalin. J. Neurosci. 22, 8251–8258 12223579 10.1523/JNEUROSCI.22-18-08251.2002 PMC6758085

[B44] Heisler L. K. Cowley M. A. Tecott L. H. Fan W. Low M. J. Smart J. L. (2002). Activation of central melanocortin pathways by fenfluramine. Science 297, 609–611 10.1126/science.1072327 12142539

[B45] Hentges S. T. (2007). Synaptic regulation of proopiomelanocortin neurons can occur distal to the arcuate nucleus. J. Neurophysiol. 97, 3298–3304 10.1152/jn.00051.2007 17360821

[B46] Hentges S. T. Nishiyama M. Overstreet L. S. Stenzel-Poore M. Williams J. T. Low M. J. (2004). GABA release from proopiomelanocortin neurons. J. Neurosci. 24, 1578–1583 10.1523/JNEUROSCI.3952-03.2004 14973227 PMC6730447

[B47] Hentges S. T. Otero-Corchon V. Pennock R. L. King C. M. Low M. J. (2009). Proopiomelanocortin expression in both GABA and glutamate neurons. J. Neurosci. 29, 13684–13690 10.1523/JNEUROSCI.3770-09.2009 19864580 PMC2785088

[B48] Herde M. K. Geist K. Campbell R. E. Herbison A. E. (2011). Gonadotropin-releasing hormone neurons extend complex highly branched dendritic trees outside the blood-brain barrier. Endocrinology 152, 3832–3841 10.1210/en.2011-1228 21791557

[B49] Heuser J. E. (1989). Review of electron microscopic evidence favouring vesicle exocytosis as the structural basis for quantal release during synaptic transmission. Q. J. Exp. Physiol. 74, 1051–1069 2560556 10.1113/expphysiol.1989.sp003333

[B50] Hill J. W. Elias C. F. Fukuda M. Williams K. W. Berglund E. D. Holland W. L. (2010). Direct insulin and leptin action on pro-opiomelanocortin neurons is required for normal glucose homeostasis and fertility. Cell Metab. 11, 286–297 10.1016/j.cmet.2010.03.002 20374961 PMC2854520

[B51] Hollopeter G. Erickson J. C. Seeley R. J. Marsh D. J. Palmiter R. D. (1998). Response of neuropeptide Y-deficient mice to feeding effectors. Regul. Pept. 75–76, 383–389. 10.1016/S0167-0115(98)00092-5 9802433

[B52] Horvath T. L. Naftolin F. Kalra S. P. Leranth C. (1992). Neuropeptide-Y innervation of beta-endorphin-containing cells in the rat mediobasal hypothalamus: a light and electron microscopic double immunostaining analysis. Endocrinology 131, 2461–2467 10.1210/en.131.3.1547 1425443

[B53] Horvath T. L. Sarman B. Garcia-Caceres C. Enriori P. J. Sotonyi P. Shanabrough M. (2010). Synaptic input organization of the melanocortin system predicts diet-induced hypothalamic reactive gliosis and obesity. Proc. Natl. Acad. Sci. U.S.A. 107, 14875–14880 10.1073/pnas.1004282107 20679202 PMC2930476

[B54] Huang L. Y. Neher E. (1996). Ca(2+)-dependent exocytosis in the somata of dorsal root ganglion neurons. Neuron 17, 135–145 10.1016/S0896-6273(00)80287-1 8755485

[B55] Hwa J. J. Ghibaudi L. Gao J. Parker E. M. (2001). Central melanocortin system modulates energy intake and expenditure of obese and lean Zucker rats. Am. J. Physiol. Regul. Integr. Comp. Physiol. 281, R444–R451 11448846 10.1152/ajpregu.2001.281.2.R444

[B56] Irani B. G. Xiang Z. Yarandi H. N. Holder J. R. Moore M. C. Bauzo R. M. (2011). Implication of the melanocortin-3 receptor in the regulation of food intake. Eur. J. Pharmacol. 660, 80–87 10.1016/j.ejphar.2010.10.101 21199647 PMC3095750

[B57] Jacobowitz D. M. O’Donohue T. L. (1978). alpha-Melanocyte stimulating hormone: immunohistochemical identification and mapping in neurons of rat brain. Proc. Natl. Acad. Sci. U.S.A. 75, 6300–6304 10.1073/pnas.75.12.6300 366617 PMC393169

[B58] Jarvie B. C. Hentges S. T. (2012). Expression of GABAergic and glutamatergic phenotypic markers in hypothalamic proopiomelanocortin neurons. J. Comp. Neurol. 520, 3863–3876 10.1002/cne.23127 22522889 PMC4114021

[B59] Jegou S. Delbende C. Tranchand-Bunel D. Leroux P. Vaudry H. (1987). alpha-Melanocyte-stimulating hormone (alpha-MSH) release from perifused rat hypothalamic slices. Brain Res. 413, 259–266 10.1016/0006-8993(87)91016-X 3607476

[B60] Joseph S. A. Michael G. J. (1988). Efferent ACTH-IR opiocortin projections from nucleus tractus solitarius: a hypothalamic deafferentation study. Peptides 9, 193–201 10.1016/0196-9781(88)90244-6 2834701

[B61] Joseph S. A. Pilcher W. H. Bennett-Clarke C. (1983). Immunocytochemical localization of ACTH perikarya in nucleus tractus solitarius: evidence for a second opiocortin neuronal system. Neurosci. Lett. 38, 221–225 10.1016/0304-3940(83)90372-5 6314185

[B62] Kaye W. H. Berrettini W. H. Gwirtsman H. E. Chretien M. Gold P. W. George D. T. (1987). Reduced cerebrospinal fluid levels of immunoreactive pro-opiomelanocortin related peptides (including beta-endorphin) in anorexia nervosa. Life Sci. 41, 2147–2155 10.1016/0024-3205(87)90533-9 2823041

[B63] Khachaturian H. Lewis M. E. Haber S. N. Akil H. Watson S. J. (1984). Proopiomelanocortin peptide immunocytochemistry in rhesus monkey brain. Brain Res. Bull. 13, 785–800 10.1016/0361-9230(84)90237-5 6099745

[B64] Kim M. S. Rossi M. Abusnana S. Sunter D. Morgan D. G. Small C. J. (2000). Hypothalamic localization of the feeding effect of agouti-related peptide and alpha-melanocyte-stimulating hormone. Diabetes 49, 177–182 10.2337/diabetes.49.5.847 10868932

[B65] King C. M. Hentges S. T. (2011). Relative number and distribution of murine hypothalamic proopiomelanocortin neurons innervating distinct target sites. PLoS ONE 6:e25864 10.1371/journal.pone.0025864 21991375 PMC3186811

[B66] Kiss J. Z. Cassell M. D. Palkovits M. (1984). Analysis of the ACTH/beta-End/alpha-MSH-immunoreactive afferent input to the hypothalamic paraventricular nucleus of rat. Brain Res. 324, 91–99 10.1016/0006-8993(84)90625-5 6097342

[B67] Kiss J. Z. Mezey E. Cassell M. D. Williams T. H. Mueller G. P. O’Donohue T. L. (1985). Topographical distribution of pro-opiomelanocortin-derived peptides (ACTH/beta-END/alpha-MSH) in the rat median eminence. Brain Res. 329, 169–176 10.1016/0006-8993(85)90522-0 2983839

[B68] Kiss J. Z. Williams T. H. (1983). ACTH-immunoreactive boutons form synaptic contacts in the hypothalamic arcuate nucleus of rat: evidence for local opiocortin connections. Brain Res. 263, 142–146 10.1016/0006-8993(83)91211-8 6301641

[B69] Kitahama K. Sallanon M. Buda C. Janin M. Dubois M. P. Jouvet M. (1986). ACTH-immunoreactive neurons and their projections in the cat forebrain. Peptides 7, 801–807 10.1016/0196-9781(86)90098-7 3025823

[B70] Krashes M. J. Koda S. Ye C. Rogan S. C. Adams A. C. Cusher D. S. (2011). Rapid, reversible activation of AgRP neurons drives feeding behavior in mice. J. Clin. Invest. 121, 1424–1428 10.1172/JCI46229 21364278 PMC3069789

[B71] Krude H. Biebermann H. Luck W. Horn R. Brabant G. Gruters A. (1998). Severe early-onset obesity, adrenal insufficiency and red hair pigmentation caused by POMC mutations in humans. Nat. Genet. 19, 155–157 10.1038/509 9620771

[B72] Lam D. D. Garfield A. S. Marston O. J. Shaw J. Heisler L. K. (2010). Brain serotonin system in the coordination of food intake and body weight. Pharmacol. Biochem. Behav. 97, 84–91 10.1016/j.pbb.2010.09.003 20837046

[B73] Lam D. D. Heisler L. K. (2007). Serotonin and energy balance: molecular mechanisms and implications for type 2 diabetes. Expert Rev. Mol. Med. 9, 1–24 10.1017/S1462399407000245 17316471

[B74] Lam D. D. Przydzial M. J. Ridley S. H. Yeo G. S. Rochford J. J. O’Rahilly S. (2008). Serotonin 5-HT2C receptor agonist promotes hypophagia via downstream activation of melanocortin 4 receptors. Endocrinology 149, 1323–1328 10.1210/en.2007-1321 18039773 PMC2275368

[B75] Lamberts R. Goldsmith P. C. (1985). Preembedding colloidal gold immunostaining of hypothalamic neurons: light and electron microscopic localization of beta-endorphin-immunoreactive perikarya. J. Histochem. Cytochem. 33, 499–507 10.1177/33.6.2582027 2582027

[B76] Lee Y. S. Challis B. G. Thompson D. A. Yeo G. S. Keogh J. M. Madonna M. E. (2006). A POMC variant implicates beta-melanocyte-stimulating hormone in the control of human energy balance. Cell Metab. 3, 135–140 10.1016/j.cmet.2006.01.006 16459314

[B77] Lee Y. S. Poh L. K. Kek B. L. Loke K. Y. (2008). Novel melanocortin 4 receptor gene mutations in severely obese children. Clin. Endocrinol. (Oxf.) 68, 529–535 10.1111/j.1365-2265.2007.03098.x 17941900

[B78] Leranth C. Williams T. H. Chretien M. Palkovits M. (1980). Ultrastructural investigation of ACTH immunoreactivity in arcuate and supraoptic nuclei of the rat. Cell Tissue Res. 210, 11–19 10.1007/BF00232137 6250704

[B79] Lim B. K. Huang K. W. Grueter B. A. Rothwell P. E. Malenka R. C. (2012). Anhedonia requires MC4R-mediated synaptic adaptations in nucleus accumbens. Nature 487, 183–189 10.1038/nature11160 22785313 PMC3397405

[B80] Liu T. Kong D. Shah B. P. Ye C. Koda S. Saunders A. (2012). Fasting activation of AgRP neurons requires NMDA receptors and involves spinogenesis and increased excitatory tone. Neuron 73, 511–522 10.1016/j.neuron.2011.11.027 22325203 PMC3278709

[B81] Low M. J. Hayward M. D. Appleyard S. M. Rubinstein M. (2003). State-dependent modulation of feeding behavior by proopiomelanocortin-derived beta-endorphin. Ann. N. Y. Acad. Sci. 994, 192–201 10.1111/j.1749-6632.2003.tb03180.x 12851316

[B82] Ludwig M. Sabatier N. Bull P. M. Landgraf R. Dayanithi G. Leng G. (2002). Intracellular calcium stores regulate activity-dependent neuropeptide release from dendrites. Nature 418, 85–89 10.1038/nature00822 12097911

[B83] MacMillan S. J. Mark M. A. Duggan A. W. (1998). The release of beta-endorphin and the neuropeptide-receptor mismatch in the brain. Brain Res. 794, 127–136 10.1016/S0006-8993(98)00223-6 9630569

[B84] Maolood N. Meister B. (2008). Dynorphin in pro-opiomelanocortin neurons of the hypothalamic arcuate nucleus. Neuroscience 154, 1121–1131 10.1016/j.neuroscience.2008.04.011 18479830

[B85] Matsumoto A. Arai Y. (1978). Morphologic evidence for intranuclear circuits in the hypothalamic arcuate nucleus. Exp. Neurol. 59, 404–412 10.1016/0014-4886(78)90232-7 648612

[B86] Meister B. (2007). Neurotransmitters in key neurons of the hypothalamus that regulate feeding behavior and body weight. Physiol. Behav. 92, 263–271 10.1016/j.physbeh.2007.05.021 17586536

[B87] Meister B. Gomuc B. Suarez E. Ishii Y. Durr K. Gillberg L. (2006). Hypothalamic proopiomelanocortin (POMC) neurons have a cholinergic phenotype. Eur. J. Neurosci. 24, 2731–2740 10.1111/j.1460-9568.2006.05157.x 17156199

[B88] Mencarelli M. Walker G. E. Maestrini S. Alberti L. Verti B. Brunani A. (2008). Sporadic mutations in melanocortin receptor 3 in morbid obese individuals. Eur. J. Hum. Genet. 16, 581–586 10.1038/sj.ejhg.5202005 18231126

[B89] Meshul C. K. Emre N. Nakamura C. M. Allen C. Donohue M. K. Buckman J. F. (1999). Time-dependent changes in striatal glutamate synapses following a 6-hydroxydopamine lesion. Neuroscience 88, 1–16 10.1016/S0306-4522(98)00189-4 10051185

[B90] Meshul C. K. McGinty J. F. (2000). Kappa opioid receptor immunoreactivity in the nucleus accumbens and caudate-putamen is primarily associated with synaptic vesicles in axons. Neuroscience 96, 91–99 10.1016/S0306-4522(99)90481-5 10683414

[B91] Meshul C. K. Stallbaumer R. K. Taylor B. Janowsky A. (1994). Haloperidol-induced morphological changes in striatum are associated with glutamate synapses. Brain Res. 648, 181–195 10.1016/0006-8993(94)91117-7 7922533

[B92] Minami M. Satoh M. (1995). Molecular biology of the opioid receptors: structures, functions and distributions. Neurosci. Res. 23, 121–145 10.1016/0168-0102(95)00933-K 8532211

[B93] Morris J. F. Pow D. V. (1991). Widespread release of peptides in the central nervous system: quantitation of tannic acid-captured exocytoses. Anat. Rec. 231, 437–445 10.1002/ar.1092310406 1793174

[B94] Morton G. J. Schwartz M. W. (2011). Leptin and the central nervous system control of glucose metabolism. Physiol. Rev. 91, 389–411 10.1152/physrev.00007.2010 21527729 PMC3379883

[B95] Mountjoy K. G. (2010). Functions for pro-opiomelanocortin-derived peptides in obesity and diabetes. Biochem. J. 428, 305–324 10.1042/BJ20091957 20504281

[B96] Mountjoy K. G. Mortrud M. T. Low M. J. Simerly R. B. Cone R. D. (1994). Localization of the melanocortin-4 receptor (MC4-R) in neuroendocrine and autonomic control circuits in the brain. Mol. Endocrinol. 8, 1298–1308 10.1210/me.8.10.1298 7854347

[B97] Murphy B. Nunes C. N. Ronan J. J. Hanaway M. Fairhurst A. M. Mellin T. N. (2000). Centrally administered MTII affects feeding, drinking, temperature, and activity in the Sprague-Dawley rat. J. Appl. Physiol. 89, 273–282 10904062 10.1152/jappl.2000.89.1.273

[B98] Murphy B. Nunes C. N. Ronan J. J. Harper C. M. Beall M. J. Hanaway M. (1998). Melanocortin mediated inhibition of feeding behavior in rats. Neuropeptides 32, 491–497 10.1016/S0143-4179(98)90077-4 9920446

[B99] Newton A. J. Hess S. Paeger L. Vogt M. C. Fleming Lascano J. Nillni E. A. (2013). AgRP innervation onto POMC neurons increases with age and is accelerated with chronic high-fat feeding in male mice. Endocrinology 154, 172–183 10.1210/en.2012-1643 23161869 PMC3529372

[B100] Nijenhuis W. A. Oosterom J. Adan R. A. (2001). AgRP(83-132) acts as an inverse agonist on the human-melanocortin-4 receptor. Mol. Endocrinol. 15, 164–171 10.1210/me.15.1.164 11145747

[B101] Norsted E. Gomuc B. Meister B. (2008). Protein components of the blood-brain barrier (BBB) in the mediobasal hypothalamus. J. Chem. Neuroanat. 36, 107–121 10.1016/j.jchemneu.2008.06.002 18602987

[B102] O’Donohue T. L. Charlton C. G. Thoa N. B. Helke C. J. Moody T. W. Pert A. (1981). Release of alpha-melanocyte stimulating hormone into rat and human cerebrospinal fluid in vivo and from rat hypothalamus slices in vitro. Peptides 2, 93–100 10.1016/S0196-9781(81)80018-6 7243627

[B103] O’Donohue T. L. Dorsa D. M. (1982). The opiomelanotropinergic neuronal and endocrine systems. Peptides 3, 353–395 10.1016/0196-9781(82)90098-5 6289277

[B104] Ollmann M. M. Wilson B. D. Yang Y. K. Kerns J. A. Chen Y. Gantz I. (1997). Antagonism of central melanocortin receptors in vitro and in vivo by agouti-related protein. Science 278, 135–138 10.1126/science.278.5335.135 9311920

[B105] Olszewski P. K. Alsio J. Schioth H. B. Levine A. S. (2011). Opioids as facilitators of feeding: can any food be rewarding? Physiol. Behav. 104, 105–110 10.1016/j.physbeh.2011.04.033 21536057

[B106] Ovesjo M. L. Gamstedt M. Collin M. Meister B. (2001). GABAergic nature of hypothalamic leptin target neurones in the ventromedial arcuate nucleus. J. Neuroendocrinol. 13, 505–516 10.1046/j.1365-2826.2001.00662.x 11412337

[B107] Padilla S. L. Carmody J. S. Zeltser L. M. (2010). Pomc-expressing progenitors give rise to antagonistic neuronal populations in hypothalamic feeding circuits. Nat. Med. 16, 403–405 10.1038/nm.2126 20348924 PMC2854504

[B108] Padilla S. L. Reef D. Zeltser L. M. (2012). Defining POMC neurons using transgenic reagents: impact of transient Pomc expression in diverse immature neuronal populations. Endocrinology 153, 1219–1231 10.1210/en.2011-1665 22166984 PMC3281533

[B109] Palkovits M. Eskay R. L. (1987). Distribution and possible origin of beta-endorphin and ACTH in discrete brainstem nuclei of rats. Neuropeptides 9, 123–137 10.1016/0143-4179(87)90051-5 3033542

[B110] Palmiter R. D. Erickson J. C. Hollopeter G. Baraban S. C. Schwartz M. W. (1998). Life without neuropeptide Y. Recent Prog. Horm. Res. 53, 163–199 9769708

[B111] Pandit R. De Jong J. W. Vanderschuren L. J. Adan R. A. (2011). Neurobiology of overeating and obesity: the role of melanocortins and beyond. Eur. J. Pharmacol. 660, 28–42 10.1016/j.ejphar.2011.01.034 21295024

[B112] Parducz A. Zsarnovszky A. Naftolin F. Horvath T. L. (2003). Estradiol affects axo-somatic contacts of neuroendocrine cells in the arcuate nucleus of adult rats. Neuroscience 117, 791–794 10.1016/S0306-4522(02)00967-3 12654332

[B113] Pennock R. L. Dicken M. S. Hentges S. T. (2012). Multiple inhibitory G-protein-coupled receptors resist acute desensitization in the presynaptic but not postsynaptic compartments of neurons. J. Neurosci. 32, 10192–10200 10.1523/JNEUROSCI.1227-12.2012 22836254 PMC3418873

[B114] Pennock R. L. Hentges S. T. (2011). Differential expression and sensitivity of presynaptic and postsynaptic opioid receptors regulating hypothalamic proopiomelanocortin neurons. J. Neurosci. 31, 281–288 10.1523/JNEUROSCI.4654-10.2011 21209213 PMC3061472

[B115] Petraschka M. Li S. Gilbert T. L. Westenbroek R. E. Bruchas M. R. Schreiber S. (2007). The absence of endogenous beta-endorphin selectively blocks phosphorylation and desensitization of mu opioid receptors following partial sciatic nerve ligation. Neuroscience 146, 1795–1807 10.1016/j.neuroscience.2007.03.029 17467916 PMC2012364

[B116] Pierce A. A. Xu A. W. (2010). De novo neurogenesis in adult hypothalamus as a compensatory mechanism to regulate energy balance. J. Neurosci. 30, 723–730 10.1523/JNEUROSCI.2479-09.2010 20071537 PMC3080014

[B117] Pinto S. Roseberry A. G. Liu H. Diano S. Shanabrough M. Cai X. (2004). Rapid rewiring of arcuate nucleus feeding circuits by leptin. Science 304, 110–115 10.1126/science.1089459 15064421

[B118] Plum L. Lin H. V. Dutia R. Tanaka J. Aizawa K. S. Matsumoto M. (2009). The obesity susceptibility gene Cpe links FoxO1 signaling in hypothalamic pro-opiomelanocortin neurons with regulation of food intake. Nat. Med. 15, 1195–1201 10.1038/nm.2026 19767734 PMC2777744

[B119] Qian S. Chen H. Weingarth D. Trumbauer M. E. Novi D. E. Guan X. (2002). Neither agouti-related protein nor neuropeptide Y is critically required for the regulation of energy homeostasis in mice. Mol. Cell. Biol. 22, 5027–5035 10.1128/MCB.22.14.5027-5035.2002 12077332 PMC139785

[B120] Qiu J. Fang Y. Ronnekleiv O. K. Kelly M. J. (2010). Leptin excites proopiomelanocortin neurons via activation of TRPC channels. J. Neurosci. 30, 1560–1565 10.1523/JNEUROSCI.5920-09.2010 20107083 PMC3095824

[B121] Qiu J. Xue C. Bosch M. A. Murphy J. G. Fan W. Ronnekleiv O. K. (2007). Serotonin 5-hydroxytryptamine2C receptor signaling in hypothalamic proopiomelanocortin neurons: role in energy homeostasis in females. Mol. Pharmacol. 72, 885–896 10.1124/mol.107.038083 17622577

[B122] Ramamoorthy P. Wang Q. Whim M. D. (2011). Cell type-dependent trafficking of neuropeptide y-containing dense core granules in CNS neurons. J. Neurosci. 31, 14783–14788 10.1523/JNEUROSCI.2933-11.2011 21994394 PMC3342306

[B123] Redrobe J. P. Dumont Y. Quirion R. (2002). Neuropeptide Y (NPY) and depression: from animal studies to the human condition. Life Sci. 71, 2921–2937 10.1016/S0024-3205(02)02159-8 12384178

[B124] Roepke T. A. Smith A. W. Ronnekleiv O. K. Kelly M. J. (2012). Serotonin 5HT2C receptor mediated inhibition of the M-current in hypothalamic POMC neurons. Am. J. Physiol. Endocrinol. Metab. 302, E1399–E1406 10.1152/ajpendo.00565.2011 22436698 PMC3378066

[B125] Roseberry A. G. Liu H. Jackson A. C. Cai X. Friedman J. M. (2004). Neuropeptide Y-mediated inhibition of proopiomelanocortin neurons in the arcuate nucleus shows enhanced desensitization in ob/ob mice. Neuron 41, 711–722 10.1016/S0896-6273(04)00074-1 15003171

[B126] Roselli-Rehfuss L. Mountjoy K. G. Robbins L. S. Mortrud M. T. Low M. J. Tatro J. B. (1993). Identification of a receptor for gamma melanotropin and other proopiomelanocortin peptides in the hypothalamus and limbic system. Proc. Natl. Acad. Sci. U.S.A. 90, 8856–8860 10.1073/pnas.90.19.8856 8415620 PMC47459

[B127] Rossi M. Kim M. S. Morgan D. G. Small C. J. Edwards C. M. Sunter D. (1998). A C-terminal fragment of Agouti-related protein increases feeding and antagonizes the effect of alpha-melanocyte stimulating hormone in vivo. Endocrinology 139, 4428–4431 10.1210/en.139.10.4428 9751529

[B128] Rowland N. E. Fakhar K. J. Robertson K. L. Haskell-Luevano C. (2010). Effect of serotonergic anorectics on food intake and induction of Fos in brain of mice with disruption of melanocortin 3 and/or 4 receptors. Pharmacol. Biochem. Behav. 97, 107–111 10.1016/j.pbb.2010.03.008 20347864 PMC2907462

[B129] Rubinstein M. Mogil J. S. Japon M. Chan E. C. Allen R. G. Low M. J. (1996). Absence of opioid stress-induced analgesia in mice lacking beta-endorphin by site-directed mutagenesis. Proc. Natl. Acad. Sci. U.S.A. 93, 3995–4000 10.1073/pnas.93.9.3995 8633004 PMC39474

[B130] Sabatier N. Caquineau C. Dayanithi G. Bull P. Douglas A. J. Guan X. M. (2003). Alpha-melanocyte-stimulating hormone stimulates oxytocin release from the dendrites of hypothalamic neurons while inhibiting oxytocin release from their terminals in the neurohypophysis. J. Neurosci. 23, 10351–10358 14614094 10.1523/JNEUROSCI.23-32-10351.2003 PMC6741015

[B131] Shutter J. R. Graham M. Kinsey A. C. Scully S. Luthy R. Stark K. L. (1997). Hypothalamic expression of ART, a novel gene related to agouti, is up-regulated in obese and diabetic mutant mice. Genes Dev. 11, 593–602 10.1101/gad.11.5.593 9119224

[B132] Smart J. L. Tolle V. Low M. J. (2006). Glucocorticoids exacerbate obesity and insulin resistance in neuron-specific proopiomelanocortin-deficient mice. J. Clin. Invest. 116, 495–505 10.1172/JCI25243 16440060 PMC1350998

[B133] Smith M. A. Hisadome K. Al-Qassab H. Heffron H. Withers D. J. Ashford M. L. (2007). Melanocortins and agouti-related protein modulate the excitability of two arcuate nucleus neuron populations by alteration of resting potassium conductances. J. Physiol. (Lond.) 578, 425–438 10.1113/jphysiol.2006.119479 17068101 PMC1864999

[B134] Sohn J. W. Xu Y. Jones J. E. Wickman K. Williams K. W. Elmquist J. K. (2011). Serotonin 2C receptor activates a distinct population of arcuate pro-opiomelanocortin neurons via TRPC channels. Neuron 71, 488–497 10.1016/j.neuron.2011.06.012 21835345 PMC3184528

[B135] Ste Marie L. Luquet S. Cole T. B. Palmiter R. D. (2005). Modulation of neuropeptide Y expression in adult mice does not affect feeding. Proc. Natl. Acad. Sci. U.S.A. 102, 18632–18637 10.1073/pnas.0509240102 16339312 PMC1309050

[B136] Sutton G. M. Duos B. Patterson L. M. Berthoud H. R. (2005). Melanocortinergic modulation of cholecystokinin-induced suppression of feeding through extracellular signal-regulated kinase signaling in rat solitary nucleus. Endocrinology 146, 3739–3747 10.1210/en.2005-0562 15961554

[B137] Thorsell A. Heilig M. (2002). Diverse functions of neuropeptide Y revealed using genetically modified animals. Neuropeptides 36, 182–193 10.1054/npep.2002.0897 12359508

[B138] Tiligada E. Wilson J. F. (1988). Ion and ion channel involvement in alpha-melanocyte-stimulating hormone secretion from superfused slices of rat hypothalamus. Neurosci. Lett. 95, 318–322 10.1016/0304-3940(88)90678-7 2852321

[B139] Tolle V. Low M. J. (2008). In vivo evidence for inverse agonism of Agouti-related peptide in the central nervous system of proopiomelanocortin-deficient mice. Diabetes 57, 86–94 10.2337/db07-0733 17909095

[B140] Tong Q. Ye C. P. Jones J. E. Elmquist J. K. Lowell B. B. (2008). Synaptic release of GABA by AgRP neurons is required for normal regulation of energy balance. Nat. Neurosci. 11, 998–1000 10.1038/nn.2167 19160495 PMC2662585

[B141] Tranchand Bunel D. Blasquez C. Delbende C. Jegou S. Vaudry H. (1989). Involvement of voltage-operated calcium channels in alpha-melanocyte-stimulating hormone (alpha-MSH) release from perifused rat hypothalamic slices. Brain Res. Mol. Brain Res. 6, 21–29 10.1016/0169-328X(89)90024-7 2549328

[B142] Tritsch N. X. Ding J. B. Sabatini B. L. (2012). Dopaminergic neurons inhibit striatal output through non-canonical release of GABA. Nature 490, 262–266 10.1038/nature11466 23034651 PMC3944587

[B143] Tsigos C. Crosby S. R. Gibson S. Young R. J. White A. (1993). Proopiomelanocortin is the predominant adrenocorticotropin-related peptide in human cerebrospinal fluid. J. Clin. Endocrinol. Metab. 76, 620–624 10.1210/jc.76.3.620 8383142

[B144] Tye K. M. Deisseroth K. (2012). Optogenetic investigation of neural circuits underlying brain disease in animal models. Nat. Rev. Neurosci. 13, 251–266 10.1038/nrm3311 22430017 PMC6682316

[B145] van den Pol A. N. (2012). Neuropeptide transmission in brain circuits. Neuron 76, 98–115 10.1016/j.neuron.2012.09.014 23040809 PMC3918222

[B146] van den Pol A. N. Cassidy J. R. (1982). The hypothalamic arcuate nucleus of rat – a quantitative Golgi analysis. J. Comp. Neurol. 204, 65–98 10.1002/cne.902040108 7056889

[B147] van Lookeren Campagne M. Oestreicher A. B. Buma P. Verkleij A. J. Gispen W. H. (1991). Ultrastructural localization of adrenocorticotrophic hormone and the phosphoprotein B-50/growth-associated protein 43 in freeze-substituted, Lowicryl HM20-embedded mesencephalic central gray substance of the rat. Neuroscience 42, 517–529 10.1016/0306-4522(91)90394-4 1716747

[B148] Veening J. G. Gerrits P. O. Barendregt H. P. (2012). Volume transmission of beta-endorphin via the cerebrospinal fluid; a review. Fluids Barriers CNS 9, 1–16 10.1186/2045-8118-9-1 22883598 PMC3439317

[B149] Vong L. Ye C. Yang Z. Choi B. Chua S. Jr. Lowell B. B. (2011). Leptin action on GABAergic neurons prevents obesity and reduces inhibitory tone to POMC neurons. Neuron 71, 142–154 10.1016/j.neuron.2011.05.028 21745644 PMC3134797

[B150] Wang Q. P. Guan J. L. Nakai Y. (1998). Beta-endorphinergic innervation of mu and delta receptor containing neurons in the dorsal raphe nucleus. Neuroreport 9, 3033–3036 10.1097/00001756-199809140-00021 9804311

[B151] Watson S. J. Richard C. W. III Barchas J. D. (1978). Adrenocorticotropin in rat brain: immunocytochemical localization in cells and axons. Science 200, 1180–1182 10.1126/science.206967 206967

[B152] Williams D. L. Kaplan J. M. Grill H. J. (2000). The role of the dorsal vagal complex and the vagus nerve in feeding effects of melanocortin-3/4 receptor stimulation. Endocrinology 141, 1332–1337 10.1210/en.141.4.1332 10746636

[B153] Williams K. W. Margatho L. O. Lee C. E. Choi M. Lee S. Scott M. M. (2010). Segregation of acute leptin and insulin effects in distinct populations of arcuate proopiomelanocortin neurons. J. Neurosci. 30, 2472–2479 10.1523/JNEUROSCI.3118-09.2010 20164331 PMC2836776

[B154] Williams K. W. Scott M. M. Elmquist J. K. (2011). Modulation of the central melanocortin system by leptin, insulin, and serotonin: co-ordinated actions in a dispersed neuronal network. Eur. J. Pharmacol. 660, 2–12 10.1016/j.ejphar.2010.11.042 21211525 PMC3085544

[B155] Wu Q. Boyle M. P. Palmiter R. D. (2009). Loss of GABAergic signaling by AgRP neurons to the parabrachial nucleus leads to starvation. Cell 137, 1225–1234 10.1016/j.cell.2009.04.022 19563755 PMC2729323

[B156] Wu Q. Clark M. S. Palmiter R. D. (2012a). Deciphering a neuronal circuit that mediates appetite. Nature 483, 594–597 10.1038/nature10899 22419158 PMC4000532

[B157] Wu Z. Xu Y. Zhu Y. Sutton A. K. Zhao R. Lowell B. B. (2012b). An obligate role of oxytocin neurons in diet induced energy expenditure. PLoS ONE 7:e45167 10.1371/journal.pone.0045167 23028821 PMC3445456

[B158] Wu Q. Howell M. P. Cowley M. A. Palmiter R. D. (2008). Starvation after AgRP neuron ablation is independent of melanocortin signaling. Proc. Natl. Acad. Sci. U.S.A. 105, 2687–2692 10.1073/pnas.0805291105 18272480 PMC2268197

[B159] Wu Q. Palmiter R. D. (2011). GABAergic signaling by AgRP neurons prevents anorexia via a melanocortin-independent mechanism. Eur. J. Pharmacol. 660, 21–27 10.1016/j.ejphar.2010.10.110 21211531 PMC3108334

[B160] Xu A. W. Kaelin C. B. Morton G. J. Ogimoto K. Stanhope K. Graham J. (2005a). Effects of hypothalamic neurodegeneration on energy balance. PLoS Biol 3:e415 10.1371/journal.pbio.0030415 16296893 PMC1287504

[B161] Xu A. W. Kaelin C. B. Takeda K. Akira S. Schwartz M. W. Barsh G. S. (2005b). PI3K integrates the action of insulin and leptin on hypothalamic neurons. J. Clin. Invest. 115, 951–958 10.1172/JCI24301 15761497 PMC1062894

[B162] Xu Y. Berglund E. D. Sohn J. W. Holland W. L. Chuang J. C. Fukuda M. (2010a). 5-HT2CRs expressed by pro-opiomelanocortin neurons regulate insulin sensitivity in liver. Nat. Neurosci. 13, 1457–1459 10.1038/nn.2664 21037584 PMC3059249

[B163] Xu Y. Jones J. E. Lauzon D. A. Anderson J. G. Balthasar N. Heisler L. K. (2010b). A serotonin and melanocortin circuit mediates D-fenfluramine anorexia. J. Neurosci. 30, 14630–14634 10.1523/JNEUROSCI.4554-09.2010 21048120 PMC3466475

[B164] Xu Y. Jones J. E. Kohno D. Williams K. W. Lee C. E. Choi M. J. (2008). 5-HT2CRs expressed by pro-opiomelanocortin neurons regulate energy homeostasis. Neuron 60, 582–589 10.1016/j.neuron.2008.09.033 19038216 PMC2631191

[B165] Yang Y. Atasoy D. Su H. H. Sternson S. M. (2011). Hunger states switch a flip-flop memory circuit via a synaptic AMPK-dependent positive feedback loop. Cell 146, 992–1003 10.1016/j.cell.2011.07.039 21925320 PMC3209501

[B166] Yaswen L. Diehl N. Brennan M. B. Hochgeschwender U. (1999). Obesity in the mouse model of pro-opiomelanocortin deficiency responds to peripheral melanocortin. Nat. Med. 5, 1066–1070 10.1038/12506 10470087

[B167] Zeltser L. M. Seeley R. J. Tschop M. H. (2012). Synaptic plasticity in neuronal circuits regulating energy balance. Nat. Neurosci. 15, 1336–1342 10.1038/nn.3219 23007188

[B168] Zhang X. van den Pol A. N. (2013). Direct inhibition of arcuate POMC neurons – a potential mechanism for the orexigenic actions of dynorphin. J. Physiol. (Lond.). [Epub ahead of print]. 10.1113/jphysiol.2012.248385 PMC3624848 23318874

[B169] Zheng H. Patterson L. M. Phifer C. B. Berthoud H. R. (2005). Brain stem melanocortinergic modulation of meal size and identification of hypothalamic POMC projections. Am. J. Physiol. Regul. Integr. Comp. Physiol. 289, R247–R258 10.1152/ajpregu.00869.2004 15746303

[B170] Zheng H. Patterson L. M. Rhodes C. J. Louis G. W. Skibicka K. P. Grill H. J. (2010). A potential role for hypothalamomedullary POMC projections in leptin-induced suppression of food intake. Am. J. Physiol. Regul. Integr. Comp. Physiol. 298, R720–R728 10.1152/ajpregu.00619.2009 20071607 PMC2838656

